# Proposition for Extending the Requirements of a Collegiate Dental Education

**Published:** 1852-10

**Authors:** B. Wood

**Affiliations:** Dentist, Nashville, Tenn.


### ARTICLE III.

*Proposition for Extending the Requirements of a Collegiate Dental Education, so as to embrace a Course of Lectures in a Medical School proper.* By B. Wood, M. D., Dentist, Nashville, Tenn.

The recent general agitation of the subject of Dental Education, through the professional journals of the day, might well suffice to arrest the attention of every dentist, and awaken in his breast hopeful anticipations for the advancement of his calling. It forms an auspicious epoch in the history of dental surgery. For whichever of the systems of instruction so ably advocated be preferable, the discussion of them has called into exercise a zeal and energy which cannot fail to eventuate in good. Thus prominently brought before the medical profession, the claims of dental surgery have been impressed upon the minds of many who had otherwise still stood aloof, as unconcerned spectators, indifferent alike to its advancement or retrogression. Individual members in our own ranks seem animated anew, and a livelier interest, nobler aims and higher hopes, appear to inspire our profession as a whole. The partial evils, such as personal ill-feeling and recrimination, arising out of the discussion of this subject, as is natural in that of any other having two sides to it, are small compared to the general good. If, however, the disputants would lose sight of their individual feelings and interests, while regarding with a single eye the welfare of our common science, it would lead to a better development and appreciation of all the great arguments in the issue.

It is not the design of this communication to take sides in the question referred to, much less to assume a hostile attitude in regard to either of the systems of dental education, the claims of which have been held up to consideration. Unquestionably each is *good* so far as it goes. If all our medical colleges were supplied with lectureships of dental surgery, and, at the same time there were a dozen special schools of dentistry prospering throughout the country, there would certainly be little cause to *mourn* about it! Nevertheless, viewing lectureships as proposed, and dental colleges as now organized, in the light of complete systems of dental instruction, either must be regarded, we think, as measurably defective. If medical schools, inoluding the branch of dental surgery in their curriculum, are to be the means of creating practical dentists, there should be additional means for this purpose, such at least as dental laboratories attached to such schools and provided with demonstrators of operative and mechanical dentistry; these should be attended by the dental students even to the omission of some of the less important medical branches: or the deficiency should be supplied by requiring a commensurate course of efficient private instruction.

But as dental colleges are already in operation, our first care would be naturally directed towards them,—to see wherein they are chiefly deficient, and in what manner this deficiency is to be remedied. If, after urging the necessary improvements,—and improvements are to be looked for from them so long as the science they teach is susceptible of improvement,—such should be neglected or found incompatible with the organization of these institutions, then it will become every lover of his profession to advocate a different organization, or the adoption of a new and more efficient system.

Impressed with the force of objections to our dental colleges, on the score of incompleteness in the department relating to general medicine, I took the liberty, upon a former occasion, to suggest the propriety of extending the term and range of their instruction so as to include a course of lectures in a regular medical school. The spirit with which the suggestion was noticed in a quarter which might be naturally expected to reflect the sentiment of faculties of dental colleges, seemed to indicate that to press the subject further would be not only unavailing

3*

but obnoxious to those having the control of these institutions.* Subsequent indications and reflections, however, have induced me to present somewhat in detail the considerations in favor of the proposed extension.

*In a letter to Dr. Blandy, published as a communication in the *Dental Times* for December, the proposition and the ground for it, were thus advanced: "Since we all admit that dental surgery is a legitimate branch of medicine, and feel the importance of medical knowledge in its practice; and since its recent extension as a science and an art, has served greatly to increase this importance, I would suggest whether it would not be well, and at the same time better satisfy the demands of the more ambitious class of dentists, and also meet the approval of medical men generally, if the dental student were required to attend *one course* of lectures in a medical school previous to his admission, or graduation in a dental college." In an allusion to this in the *Dental Register*, edited by the Prof. of Principles and Practice of the Ohio Dental College, the following remarks occur: "Mr. Wood suggests that to 'satisfy the demands of the more ambitious class of dentists, and also meet the approval of medical men generally,' the dental student be required to attend," &c. (It will be observed that the suggestion is here *denied the benefit of the very basis upon which it is grounded*, although this denial necessitated the dismemberment of a sentence, the parts of which were intimately connected, while the *incidental advantages* are made to appear as *the* basis!) The editor continues: "We scarce know whether to regard this as a compliment to dental colleges or not. The inference is, however, that dental schools are not capable of giving proper preparatory instruction. The fact is, that many in the profession appear to be but little acquainted with the course of instruction given in our dental colleges. Or if they are, they have the same feeling with regard to medical schools, which the public formerly had with reference to French dentists. If he [French dentists,] hailed from France, and was addressed as *Monsieur*, it was evidence of dental qualification. We rejoice that the profession has outlived this, and hope it may survive the other, and come to realize that she [it, the profession,] has within herself all that which is necessary to ennoble and sustain herself"—(*Reg. Jan.* 1852, p. 123.) That the editor, a medical man of standing sufficient to claim for him an *honorary degree* in medicine, should *deride* medical colleges is remarkable, since, if not experimentally "acquainted with the course of instruction given" in them, still his knowledge of the branches they teach should suffice to claim for them some little respect, even as sources of instruction for dental students. Professors who thus deride medical schools, and who, moreover, have obtained their qualifications and their honors, both in medicine and dentistry, without putting themselves to the trouble of a collegiate course in either, should not grumble that "many appear to be but little acquainted" with *their* colleges!

In the same connection the editor quotes from "one other remark of Mr. Wood's," wherein he seems to find an imputation of failure in regard to the Ohio college, in rebutting which, the words "opposition and machinations" are

This proposition is offered upon the belief that dental surgery is, and of right ought to be, a department of general medicine; that a knowledge of the latter is the true basis of correct practice in the former; that dental practitioners should be medical men, created from, and belonging to the regular profession; that the student in this specialty must be in reality, (though not of necessity *nominally*,) a doctor of general medicine before he can be truly a doctor of dental surgery, and, that a course of collegiate dental education should be such as *virtually* to entitle him to the honor of both.

Dental surgery is generally conceded to be a medical science. It is so styled and treated of by the most eminent in our profession. It cannot be supposed that this would be done merely for the purpose of exalting the art in the eyes of the mass to a rank which it does not deserve, but rather from a conscientious conviction in regard to its true character and position. That this position is taken by men of high standing, whose attainments, both in dentistry and general medicine, qualify them to form a just opinion, and entitle that opinion to confidence, and not by charlatans and impostors, anxious to magnify their calling as a means of self-aggrandizement, might furnish a sufficient guarantee for its reception. It has moreover been too strongly fortified by physiological and pathological facts and indications to admit of question. To *argue* the point would require an undue latitude of discussion, involving not only a survey of the general principles, and many of the details, of this specialty, but extending in like manner into the so ambiguously employed as to leave a doubt whether a personal application of them was intended or not; but as no such imputation was made in the remark, or implied, or intended to be implied, but instead of this quite the contrary, as the quotation mark, including the word "failure," the connection of the sentence, and the whole tenor of the remarks alluded to will show, I need not give it any further notice.

If all recommendations for the establishment and improvement of dental colleges are to be regarded in the light of evils, such as "the profession has outlived," and as it is to be hoped "it may survive," and of opposition and machinations designed for the overthrow of these schools, thus serving no purpose but to afford occasion for the rallying cry of persecution, then it were about as well to "hang up the fiddle and the bow," and wait for better times.

various branches of medicine,—a task which, it is believed, the concurrent opinion of the entire dental profession renders superfluous. I shall receive it as valid in its full sense.

This position being assumed, it leads to another, namely:— That a knowledge of medicine is necessary in the practice of dental surgery, as well as in any other specialty. This position also admits of ample confirmation by an appeal to the physiology, pathology and therapeutics of the dental organs and their appendages, and to facts and cases daily occurring in dental practice. The subject has been abundantly elucidated in dental books and periodicals, and its recent discussion certainly leaves but little further to advance.

The mouth,—the appropriate sphere of the dentist's care,— is subject to the forms of disease common to other parts of the system, influencing, and being influenced by the condition of the latter; while it is also liable to affections peculiar to itself. If to plug or extract a tooth, or construct an artificial substitute, constituted the sum of all the duties and responsibilities of the dentist, and of all that *ought* to be expected and required of him, then, perhaps, there would be less need of a knowledge of medicine on his part; for then the physician would be required to look to the welfare of the mouth, sending his patients, as necessity called for, to the mechanical dental operator,— making use of him in very nearly the same way the surgeon does of the manufacturer of braces, splints, artificial limbs, or the oculist of the maker of artificial eyes, &c. But regarding *the mouth* as the legitimate province of the dentist, the case is clearly different. And can we claim less? Do not the teeth themselves give constant rise to diseases of the neighboring parts, and does it not become the practitioner to know whether these are the result of dental affections or of other causes, and to govern his treatment accordingly? And do they not moreover give rise to constitutional disturbances, demanding general remedies? The treatment of the teeth, however restricted, becomes, after all, the surgery of the mouth; and this is nothing less than saying, in its broadest sense, having to do with a most important and delicate part of the human system: the sympathies are nowhere more extensive and complicated than here, and the treatment nowhere more loudly call for all the light to be derived from a knowledge of general principles.

No one will deny that in the practice of surgery, shorn of the "dental," a knowledge of medicine is indispensable. It is a settled conviction with medical men that "no one can ever lay just claim even to the title of surgeon, far less hope for eminence or success, unless he be equally qualified to assume both the appellation and the employment of the physician." And if dental surgery is entitled to, or is ever to assume the rank we claim for it, a like qualification is no less indispensable on the part of its practitioners. Such is indeed the prevailing belief of the best informed dentists of the day, whatever system of education they have chosen to advocate, as could be abundantly shown by a reference to their writings. Nor would there seem to be room for a different opinion when we consider the fact, that constitutional treatment is frequently called for in the ordinary routine of dental practice as limited exclusively to operations upon the teeth.

It is, then, claimed as beyond dispute, that a knowledge of general medicine *is* necessary in the practice of dental surgery.

With these views respecting the qualification required in dental surgery, the question arises, do dental colleges, as now organized, afford an adequate preparation for its practice? Is their range of medical instruction sufficient for the creation of scientific and reliable practitioners in this specialty? The title and tenor of this paper assumes the negative. Admitting them to be good as far as they go, and as efficiently conducted as may be, yet their requirements would seem to be, of necessity, inadequate to the great end in view. Others may entertain a contrary opinion, but surely not those who regard our science in its true light and full bearings. Few would pretend that the course in medical colleges is *abundantly* sufficient to qualify the physician for his duties, much less that that pursued in dental schools can perfect the student in both medicine and dentistry. I would not derogate dental schools, for which, as instruments for the advancement of a noble science, I have ever cherished friendly feelings and ardent hopes, but I must be permitted, at all hazards, to indicate the appreciation I entertain as to the nature and demands of that science; and I would have the requirements of the former commensurate with those of the latter.

The medical fraternity and the community generally, are clamorous for extension of the range and term of instruction in schools of medicine;—(and it is not accounted as evidence of "hostility;")—this clamor comes mostly from the active friends of these institutions. And would it not seem that in our own profession, the friends of dental colleges, actuated by the same spirit, should examine the system of professional education adopted in them, and, if found defective, advocate its extension? We say the "*friends*" of dental colleges ought to interest themselves in this matter. In a late circular of one of these schools it is said, "they are mainly indebted to" their "enemies" for having been brought prominently before the public; and it were a pity they should be under obligation to their *enemies* for all things tending to their advancement! The true way to promote the permanent prosperity of such institutions, is to recognize whatever defects may exist, to ascertain whatever abuses may be connected with them, and to manifest an earnest endeavor to correct the evils.

Let us inquire, then, what are the requirements of dental colleges. As that at Baltimore may be regarded the model as well as pioneer of the rest, the others differing little in their organization from this, it may be taken as the criterion.

The collegiate term consists of two courses of theoretical and practical instruction of four months each. A course of lectures in a medical school, or four years reputable dental practice, is taken as equivalent to a course in the college. No private tuition is required, differing in this respect from medical colleges.*

* The Philadelphia College, which is to go into operation in November, 1852, will afford an exception in the above particular, one of its requisites for graduation being, that the student "must have studied under a private preceptor at least two years, including his course of instruction in the college." This is an

Thus the term of pupilage for the completion of a dental education would be comprised in eight months, or with a previous medical course, in four months. Will it be asserted that this length of time is sufficient to create safe and efficient operators in the different branches of dentistry? Can any dentist who understands the nature of his duties be convinced that it is, even if the whole time were devoted to practical exercises? But waiving this for the present, what is the range of theoretical studies, including those relating to medicine and surgery?

"The Faculty of the Baltimore College consists of a
Professor of Principles and Practice of Dental Surgery.
" Operative and Mechanical Dentistry.
" Special Pathology and Therapeutics.
" Anatomy and Physiology.
Demonstrator of Mechanical Dentistry and Lecturer on Chemistry.

Three of the above offices are filled by practicing dentists, and two (special anatomy and physiology,) by physicians.—Lectures in the four departments, named above, are delivered each afternoon. The mornings are especially devoted to the *practice* of the various branches."*

Thus one-half of the time is occupied with practical instruction, and the balance is devoted to the principles of medicine and dentistry. Now taking dental surgery in its various bearings, so as to include what might rightly be expected in a dental college beyond what is taught in medical schools,—such as the pathology and therapeutics of the dental system, the surgical and medical treatment and the remedial agents brought into requisition; the anatomy of the teeth, with appropriate illustrations drawn from comparative anatomy; their physiology and its relation to hygiene; dental physiognomy, involving the principles of æsthetics, or of beauty and proportion, so far at auspicious advance, and speaks well for the determination of the faculty of this infant enterprise, who it is hoped will be encouraged and sustained in the bold stand they have thus early assumed.

* Amer. Jour. Dent. Science, New Series, vol. 2, page 69.

least as connected with the expression of the face; the sciences of metallurgy and mechanics as applicable to mechanical dentistry,—and one would naturally conclude there would be but a small moiety of time left for inculcating the principles of medicine and surgery proper. Even anatomy and physiology would hardly be expected to extend beyond the region of "the face and neck" at most. As to the other branches taught in an ordinary medical course, they are left out of the arrangement. But suppose the time allotted for the inculcation of theoretical principles, *i. e.* one-half of the whole term, be equally divided between medicine and dentistry; then, upon the one hand, these schools present the spectacle of teaching all the medical knowledge adjudged necessary for the dentist, in the space of two months, or, if a practitioner, he is put through in *one month.* On the other hand the student of medicine is made a proficient in operative dentistry by a practical training of two months.

Now I put it to the conscience of every well-informed dentist to say if the above range of instruction meets the just wants and demands of the dental profession? If so, why all this tumult and denunciation against dentists who profess to initiate their office-students into all the mysteries of the art in the space of "a few months, or even a few weeks?" Why so much outcry in regard to the project of erecting a chair in medical colleges for the purpose of enabling these to teach dental surgery? If it is said that *evidence* of proficiency is required in order to obtain the honors of the institution, and in default therein, additional instruction must be had by a longer probation, either in the college or under a preceptor, or through dint of self-training, it may be replied, that the above is all the tuition demanded in the conditions of graduation; the belief is inculcated that proficiency is attainable by the course indicated; and to require more at the examinations than at the colleges, were manifestly unjust as inducing the honor of a degree to be sought in a means which, upon trial, shall prove inadequate for obtaining it. It is certain medical schools do not require, in the examinations, greater evidence of qualification than can be easily enough acquired in their prescribed course of study. Nor is it likely dental colleges should afford an exception in this respect. Their prescribed course of instruction, as above described, must then be taken as the sum of their requirements; and it needs but a glance to be convinced of its inadequacy.

But if, conceding the point at issue, it is said that the great object contemplated by these institutions is to afford such additional facilities as may enable the man of general acquirements the better to perfect himself as a practitioner, or to teach such scientific principles and correct modes of operating as shall furnish beginners with a guide by which they may work out their own proficiency; then, why not so declare it at once; and thus admit that there *is* "room for improvement," instead of necessitating a resort to a prolix detail of statistics, facts, and arguments to *prove* it! Why claim to make thoroughly educated dentists, medical and operative, in the college halls? Why publish it to the world that the course there pursued is "*ample and sufficient*" for this? Why essay to propagate the idea that a dental diploma is *the* guarantee of competency—the "*only* passport to patronage?" Why proclaim in professional journals, that, without such acknowledgment of a "regular" education, a dentist "can never rise to any thing like respectability and standing in the profession and in the community," that, "he is still a quack, and can never transfer himself from the class and standing of a quack to those of a man of science?"*

Now I am not going to quarrel with professors for indulging in such strains occasionally, but I think it right to express the opinion that it does not "help the cause" much. It may have led some to "secure" their "passport," but it is likely they "hurried" to *societies* for it, rather than into the colleges!

It is evident that too much has been claimed on the part of dental colleges for their means, while at the same time great injustice has been done to many in the profession, who, like most of those now constituting dental faculties, never had the advantages of what is termed a "regular dental education." The good of these institutions require that they abandon a plan

* Amer. Jour. Dent. Science, vol. 9, page 6.

of procedure, which, though happily provoked by the apathy and hostility of a few narrow-minded and selfish dentists, yet, being indiscriminate in its bearings, is no doubt calculated greatly to weaken the general feeling of sympathy and respect towards them. But to return from this digression: that the present system of collegiate dental education is greatly inadequate to the wants of the profession is virtually admitted by its most zealous advocates. Prof. Harris, with a candor indicative of a return to the right spirit, says, in reply to objections on this score, that if dental colleges were better patronized, "they would *increase their professorships*," &c.; and from the last Annual Announcement of the Baltimore College, it appears that its range of instruction has been extended by the creation of a distinct chair of Chemistry; it is there also recommended, that "private instruction of the right kind is valuable, and *should always be obtained if possible.*" Of course additional professorships, and private tuition would not be needed if the present means were amply sufficient. Again, in a later article, Dr. Harris says: "if it could be done, we would require every student of dental surgery to graduate both in medicine and dentistry." Now this, it is believed, is just what is needed to make our profession what it should be, and if so, it should be the ultimate aim and object of every one who would advance the cause of dental education. To carry it out at once, to its full extent, might, perhaps, be impracticable; but this need not preclude all hope of final success, nor prevent the endeavor to make some progress towards it.

( To be Continued. )

